# Neuropathological changes in dorsal root ganglia induced by pyridoxine in dogs

**DOI:** 10.1186/s12868-020-00559-3

**Published:** 2020-03-24

**Authors:** Sumin Yun, Woosuk Kim, Min Soo Kang, Tae Hyeong Kim, Yoonhwan Kim, Jin-Ok Ahn, Jung Hoon Choi, In Koo Hwang, Jin-Young Chung

**Affiliations:** 1 Department of Veterinary Internal Medicine and Institute of Veterinary Science, College of Veterinary Medicine, Kangwon National University, 1 Kangwondaehak-gil, Chuncheon-si, Gangwon-do 24341 South Korea; 2 Department of Anatomy and Cell Biology, College of Veterinary Medicine, and Research Institute for Veterinary Science, Seoul National University, 1 Gwanak-ro, Gwanakgu, Seoul, 08826 South Korea; 3 Department of Veterinary Anatomy, College of Veterinary Medicine, Kangwon National University, 1 Kangwondaehak-gil, Chuncheon-si, Gangwon-do 24341 South Korea

**Keywords:** Dog, Dorsal root ganglia, H-reflex, Pyridoxine, Sensory neuropathy

## Abstract

**Background:**

Pyridoxine (PDX; vitamin B_6_), is an essential vitamin. PDX deficiency induces various symptoms, and when PDX is misused it acts as a neurotoxicant, inducing severe sensory neuropathy.

**Results:**

To assess the possibility of creating a reversible sensory neuropathy model using dogs, 150 mg/kg of PDX was injected subcutaneously into dogs for 7 days and body weight measurements, postural reaction assessments, and electrophysiological recordings were obtained. In addition, the morphology of dorsal root ganglia (DRG) and changes in glial fibrillary acidic protein (GFAP) immunoreactive satellite glial cells and ionized calcium-binding adapter molecule 1 (Iba-1) immunoreactive microglia/macrophages were assessed at 1 day, 1 week, and 4 weeks after the last PDX treatment. During the administration period, body weight and proprioceptive losses occurred. One day after the last PDX treatment, electrophysiological recordings showed the absence of the H-reflex in the treated dogs. These phenomena persisted over the four post-treatment weeks, with the exception of body weight which recovered to the pre-treatment level. Staining (CV and HE) results revealed significant losses of large-sized neurons in the DRG at 1 day and 1 week after PDX treatment cessation, but the losses were recovered at 4 weeks post-treatment. The Iba-1 and GFAP immunohistochemistry results showed pronounced increases in reactive microglia/macrophage and satellite glial cell at 1 day and 1 week, respectively, after the last PDX treatment, and thereafter, immunoreactivity decreased with increasing time after PDX treatment.

**Conclusions:**

The results suggest that PDX-induced neuropathy is reversible in dogs; thus, dogs can be considered a good experimental model for research on neuropathy.

## Background

Pyridoxine (PDX), along with pyridoxal and pyridoxamine, are compounds that can be referred to as vitamin B6. PDX is considered an essential vitamin, and a PDX deficiency induces various symptoms [1]. However, PDX has also been identified as a neurotoxicant that can induce severe sensory neuropathy in response to chronic abuse of oral PDX supplements [2]. Compelling evidence related to the functional and physiological parameters induced by PDX neurotoxicity has been demonstrated in rodents [3–6], dogs [7–9], and humans [10].

There are many disorders that involve invasion of peripheral nerves, inducing sensory neuropathy, which is caused by genetic diseases, metabolic imbalance, endocrine disease, toxins, fluoroquinolone toxicity, inflammation, and physical trauma. Accordingly, many clinical trials have been conducted to develop a treatment for sensory neuropathy; however, it is apparent that more animal models of sensory neuropathy are required [11–13]. Dogs are considered valuable animal models because they are more similar to humans than rodents. However, fewer neurological studies have been conducted using canine models than rodent models [7].

All sensory axons pass into the dorsal root ganglion (DRG) and then into the spinal cord. Therefore, observation of the DRG is useful when attempting to obtain information about sensory tracts [14].

In this study, we conducted neurological examinations and observed H-reflexes and chronological changes in both satellite glial cells (SGC) and microglia/macrophages using glial fibrillary acidic protein (GFAP) and ionized calcium-binding adapter molecule 1 (Iba-1), respectively, in the DRG. Specifically, we evaluated the H-reflex and expressions of GFAP and Iba-1 to determine if they changed with time in response to the subcutaneous injection of PDX for 7 days. To accomplish this, a dog model of sensory neuropathy was established by administering a subcutaneous injection of PDX over a short period. We observed the dogs for 4 weeks after the last PDX treatment to identify post-treatment chronological changes in the effects of PDX on the DRG.

## Results

### Body weight measurements

**Fig. 1:**
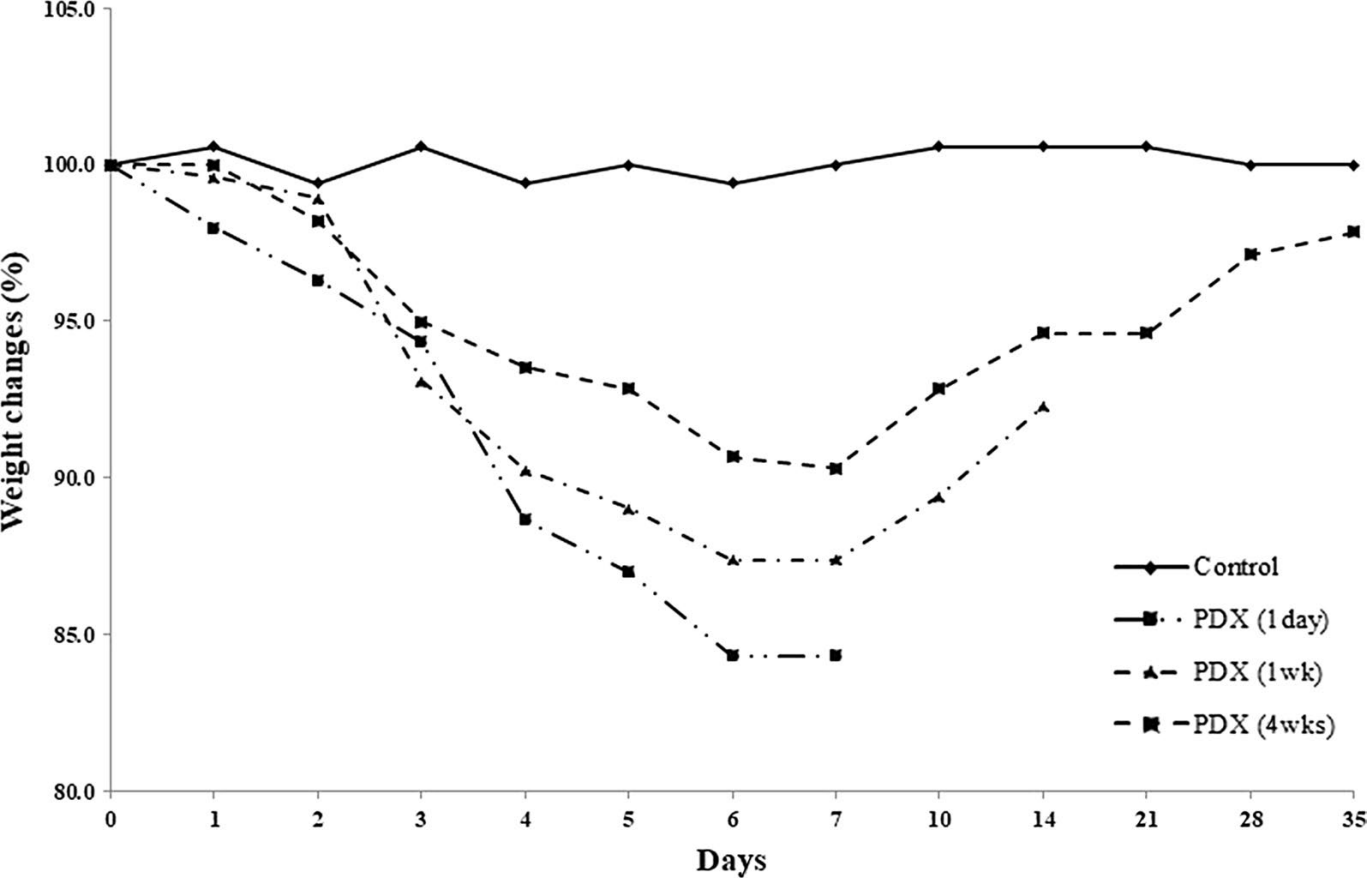
Relative body weight changes in control and PDX-treated groups. The weight changes are expressed by percentages relative to that at day 0 of treatment. Body weight decreased 1 day after the last PDX treatment in the PDX-treated group

Weight loss was observed in the PDX-treated group (Fig. 1) with a significant relative decrease from the body weight before PDX treatment (100% ± 0%) to that after the last PDX treatment (87.3% ± 3.8%) (*P *< 0.0001). However, there was no significant difference in the relative body weight before PDX treatment (100 ± 0%) and that at 4 weeks after the last PDX treatment (97.9% ± 4.8%) (*P* = 0.6579).

### Postural reaction assessments

All dogs in the control group showed normal responses to the postural reaction test stimuli. However, all dogs in the PDX-treated group displayed evidence of neurologic disorders. On days 3 or 4 of the 7 days of PDX injection, proprioceptive abnormalities involving the hindquarters were observed during postural reaction testing. One day after the last PDX treatment, all dogs exhibited hindlimb stiffness when attempting to walk or turn. These symptoms were maintained at 1 week after the last PDX treatment. At the end of the experimental period (4 weeks after the last PDX treatment), there was no evidence of hindlimb stiffness, but proprioceptive abnormalities in the hindquarters remained.

### Electrophysiological recording

**Fig. 2:**
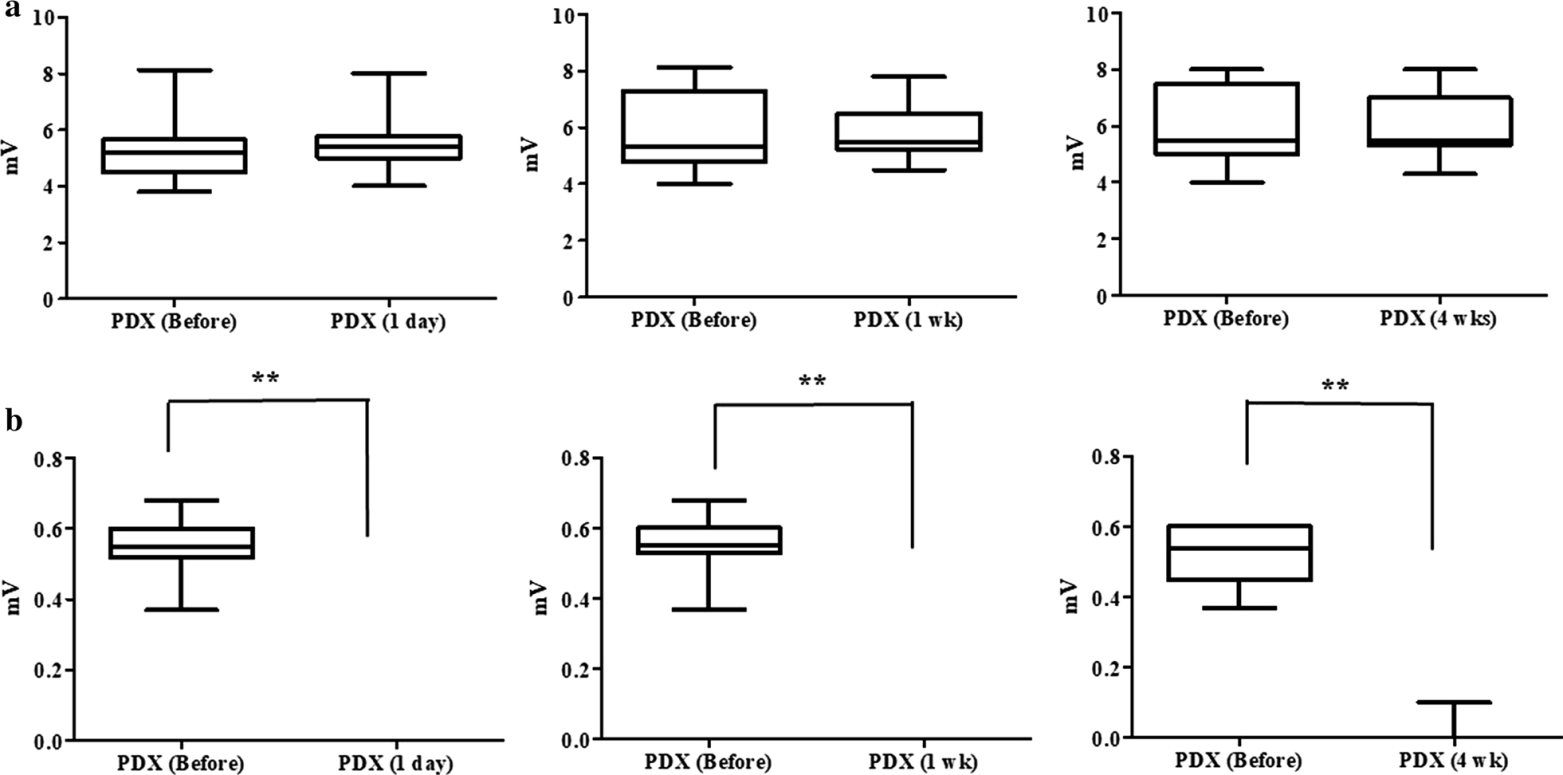
Electrophysiological recording. There were no significant differences in M wave amplitudes (**a**); however, compared to the before PDX treatment amplitudes, there were significant differences in the H-reflex amplitude at 1 day, 1 week, and 4 weeks after the last PDX treatment (**b**). **P < 0.05 indicates a significant difference. The bars indicate mean ± SEM values

Evaluation of the direct-evoked muscle potentials (DEMP; M wave) and the reflex-evoked muscle potentials (REMP; H-reflex) in the clinically normal control dogs showed that mean M wave amplitudes (5.4 ± 2.3 mV) were much higher than the mean H-reflex amplitudes (0.5 ± 0.3 mV). Examination of the mean M wave latencies (2.8 ± 0.3 ms) showed that the M wave was an early response whereas the mean H-reflex latencies (16.2 ± 2.5 ms) showed that the H-reflex was a late response [6]. Compared to the M wave amplitude before PDX treatment, there were no significant changes in M wave amplitudes in the PDX-treated group at 1 day (5.6 ± 0.9 mV), 1 week (5.8 ± 1.3 mV), or 4 weeks (5.9 ± 0.9 mV) after the last PDX treatment (*P *> 0.05) (Fig. 2a). However, compared with the before PDX treatment amplitude, there were significant changes in the H-reflex amplitude at 1 day, 1 week, and 4 weeks after the last PDX treatment (*P *< 0.05) (Fig. 2b). At 1 day after the end of PDX treatment, there was no H-reflex detected among the PDX-treated dogs, and that condition was sustained until 4 weeks after the last PDX treatment. Interestingly, among the PDX-treated dogs, one dog presented two H-reflex episodes among the eight measurement periods.

### Immunohistochemistry

**Fig. 3:**
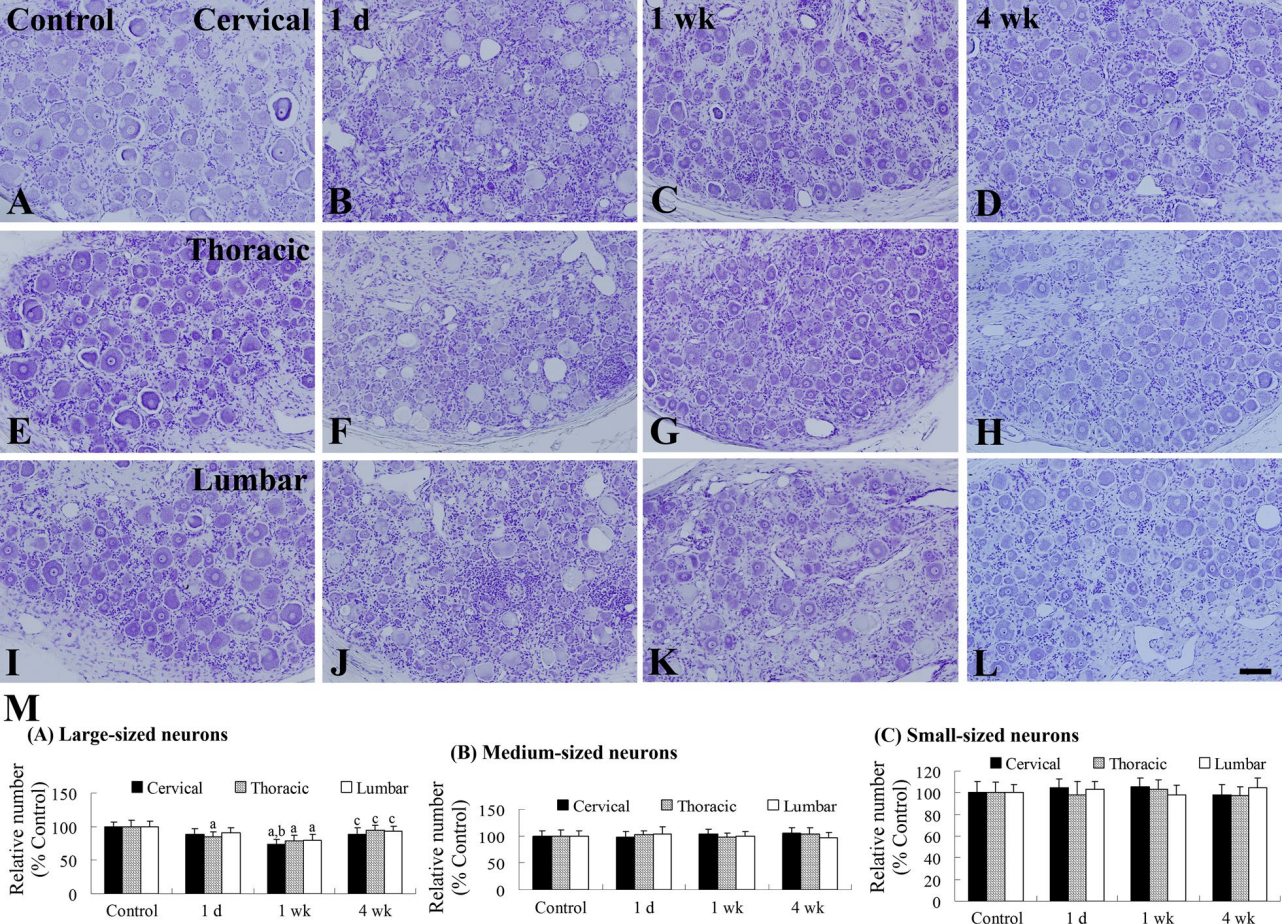
Cresyl violet (CV) staining of the DRG in control (**a**, **e**, **i**) and PDX-treated (**b**–**d**, **f**–**h**, **j**–**l**) groups. In the control group, neurons in all DRG regions were well-stained with CV. Note that there was a decrease in CV-stained large-sized neurons in all regions of the DRGs at 0 and 1 weeks after the last PDX treatment. Scale bar = 100 μm. M: Relative numerical analysis of CV-stained neurons according to neuron size (*n* = 2–3 per group; ^a^*P* < 0.05, significantly different from the control group; ^b^*P* < 0.05, significantly different from the 1 day after the last PDX treatment group; ^c^*P* < 0.05, significantly different from the 1 week after the last PDX treatment group). The bars indicate mean ± SEM values

Among the control dogs, neurons were abundant in all DRG regions based on cresyl violet (CV) (Fig. 3a, e, i) and hematoxylin and eosin (HE) (Additional file 1: Figure S1A, E, I) staining results. At 1 day after the last PDX treatment, the numbers of CV- and HE-stained large-sized neurons were significantly low in the cervical, thoracic, and lumbar DRG regions (Fig. 3m), while the number of CV- and HE-stained small- and medium-sized neurons did not change. In addition, there were no significant differences in the amount of neuronal damage among the three DRG regions (Fig. 3b, f, j, m). However, many HE-stained nuclei were observed in all DRG regions (Additional file 1: Figure S1B, F, J).

At 1 week after the last PDX treatment, the number of CV-stained large-sized neurons was significantly higher in all DRG regions compared to that in the PDX-treated group at 1 day after the last PDX treatment and was approximately 77.3% of the number in the control group (Fig. 3c, g, k, m). In addition, HE staining in the PDX-treated group revealed that some hematoxylin-stained nuclei although aggregated in the cervical DRG region (Additional file 1: Figure S1C) were not present in aggregates in the thoracic and lumbar regions (Additional file 1: Figure S1G, K). At 4 weeks after the last PDX treatment, the number of CV-stained large-sized neurons was higher in all DRG regions compared to that in the PDX-treated group at day 1 or week 1 after the last PDX treatment; moreover, about 92.3% of the number of neurons in the control group were observed in the PDX-treated group (Fig. 3d, h, l, m). In addition, HE staining in this group showed a lower level of aggregated nuclei stained with hematoxylin in all DRG regions (Additional file 1: Figure S1D, H, L).

**Fig. 4:**
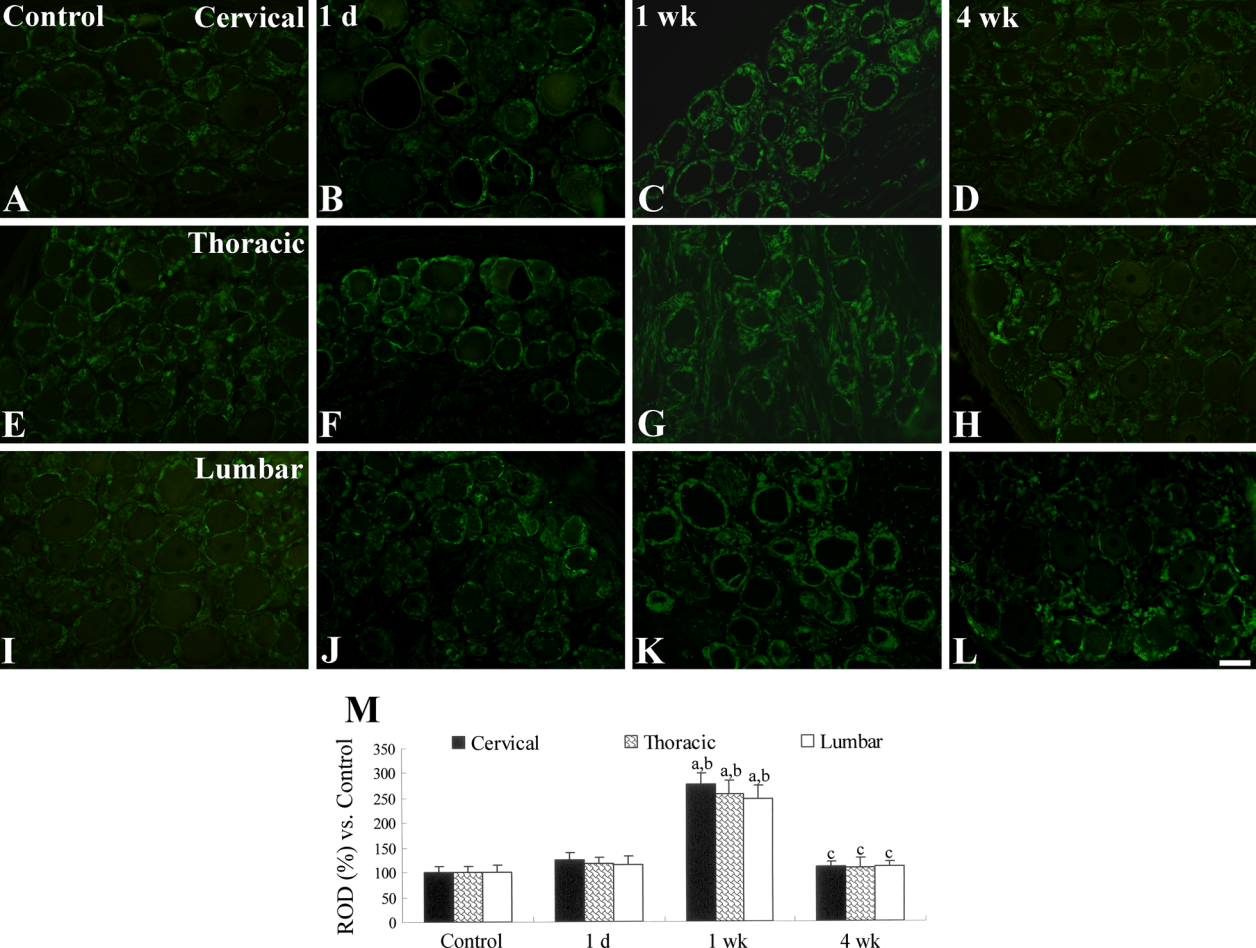
GFAP immunostaining of the DRG in control (**a**, **e**, **i**) and PDX-treated (**b**–**d**, **f**–**h**, **j**–**l**) groups. In the control group, GFAP immunoreactivity is shown in the DRG and GFAP immunoreactive satellite glial cells are abundant at 1 week after the last PDX treatment, after which the abundance decreased with time. Scale bar = 100 μm. M: Relative optical density (ROD; as a percentage) of each DRG region of the control group (*n* = 2–3 per group; ^a^*P* < 0.05, significantly different from the control group; ^b^*P* < 0.05, significantly different from the 1 day after the last PDX treatment group; ^c^*P* < 0.05, significantly different from the 1 week after the last PDX treatment group). The bars indicate mean ± SEM values

GFAP immunoreactive SGCs were detected in all DRG regions in the control group, and the SGCs surrounded the neurons in the DRG (Fig. 4a, e, i). One day after the last PDX treatment, GFAP immunoreactivity was slightly, but not significantly, increased in the DRG (Fig. 4b, f, j, m), but at 1 week after PDX treatment and compared to the control group, GFAP immunoreactivity was significantly increased in the cervical, thoracic and lumbar regions of the DRG (Fig. 4c, g, k, m). At 4 weeks after PDX treatment, GFAP immunoreactivity decreased significantly in the regions of the DRG and was similar to the levels in the control group DRG regions (Fig. 4d, h, l, m).

**Fig. 5:**
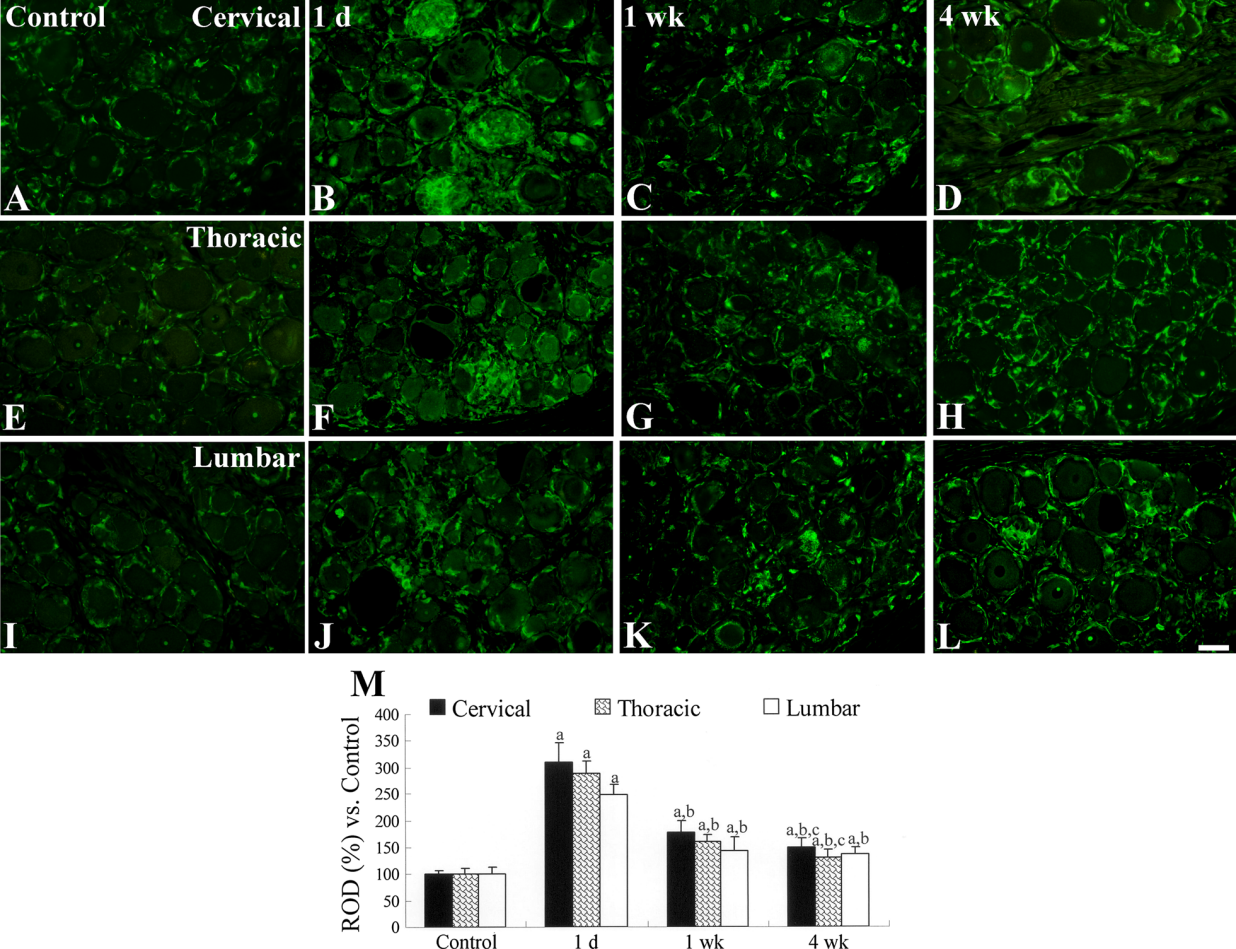
Iba-1 immunostaining of the DRG in control (**a**, **e**, **i**) and PDX-treated (**b**–**d**, **f**–**h**, **j**–**l**) groups. In the control group, Iba-1 immunoreactivity is present in the DRG and microglial Iba-1 immunoreactivity in the DRG peaked 1 day after PDX treatment, after which they decreased. Scale bar = 100 μm. M: Relative optical density (ROD; as a percentage) of each DRG region in the control group (*n* = 2–3 per group; ^a^*P* < 0.05, significantly different from the control group; ^b^*P* < 0.05, significantly different from the 1 day after the last PDX treatment group; ^c^*P* < 0.05, significantly different from the 1 week after the last PDX treatment group). The bars indicate mean ± SEM values

In the control group, ionized calcium-binding adapter molecule 1 (Iba-1) immunoreactive microglia/macrophages were detected in all DRG regions (Fig. 5a, e, i). At 1 day after the last PDX treatment, the Iba-1 immunoreactivity in the PDX-treated group had significantly increased in the DRG and the Iba-1 immunoreactive microglia/macrophages exhibited a hypertrophied cytoplasm and a round shape (Fig. 5b, f, j, m). At 1 and 4 weeks after the last PDX treatment, Iba-1 immunoreactivity had gradually decreased in the DRG, but Iba-1 immunoreactivities at these times were higher than those in the DRG of the control group (Fig. 5c, d, g, h, k, l, m).

## Discussion

PDX is a well-known agent against peripheral neuropathy and is not reported to be harmful to other organs [15–17]. Several studies have demonstrated that PDX causes neuronal death in the cell bodies and long myelinated fibers of DRGs in human, rat, and dog [3–10]. A rat model administered with 800 mg/kg/mL of pyridoxine via an intraperitoneal route produced a significant decrease in large-sized cells [18] and, in that study, the H-reflex was measured to demonstrate that PDX could induce sensory neuropathy. In contrast, a dog model treated with 150 mg/kg PDX subcutaneously, and in which electrophysiological recordings were taken during peripheral nerve stimulation in the experimental group, showed results consistent with the presence of selective toxicity to sensory nerve function, but not motor nerve function [7]. In the present study, we selected PDX for the induction of peripheral neuropathy in dogs because neuropathy in a dog model can be induced by a low dosage of PDX compared to that in a rat model. In this study, we investigated the effect of PDX on cell morphology, SGCs, and microglia/macrophages at 0, 1, and 4 weeks after the last PDX treatment in dogs to assess the possibility of using dogs as a reversible animal model for PDX-induced neuropathy.

Based on previous studies [7, 8], we were concerned that the relationship with time of changes in neurological examination, electrophysiological recording, and histopathological analysis results of a dog model of sensory neuropathies produced by PDX over a short period. In previous experiments, body weights decreased during the PDX administration period, which was related to changes in food consumption. A similar phenomenon occurred during this study’s PDX administration period, but body weight did increase with time after the PDX administration period ended. We also observed that the H-reflex disappeared during the 4 weeks after the last PDX treatment, even though histological results showed that the DRG had recovered. Based on these results, we speculate that structural recovery of the DRG occurs before its functional recovery.

In this study, we also observed chronological changes in SGCs and microglia, cells that contribute to several neuropathological conditions [19, 20]. GFAP immunoreactivity in SGCs was significantly upregulated at 1 week after the last PDX treatment and decreased by 4 weeks after the last PDX treatment. These results are similar to previous findings in which GFAP expression was upregulated in SGCs of an injured DRG from day 3 onward immediately after sciatic nerve injury. In addition, proprioceptive abnormalities remained at 3–4 days after PDX treatment.

Iba-1 immunoreactivity of microglia was significantly increased 1 day after the last PDX treatment and microglial Iba-1 immunoreactivity decreased throughout the rest of the experimental period. This result was supported by our histological results showing that neuronal death was prominent at 1 day after the last PDX treatment but the number of large-sized neurons in the DRG increased thereafter. Activation of microglia/macrophages may be associated with the initiation and maintenance of mechanical hypersensitivity [21]. In a diabetic neuropathy model, Iba-1 immunoreactive macrophages are significantly increased in the lumbar DRG region at 1 week after streptozotocin treatment [22]. Similarly, in a sciatic nerve injury model, macrophages gradually increase until 7 days after injury [23].

## Conclusions

We have established a reversible dog model of sensory neuropathy by administering subcutaneous injections of PDX over a short period (7 days). Recovery was observed over a 4-week period after the last PDX treatment. Further studies into the mechanism of reversible sensory neuropathy created by PDX in dogs are necessary.

## Methods

### Animal model

**Fig. 6:**
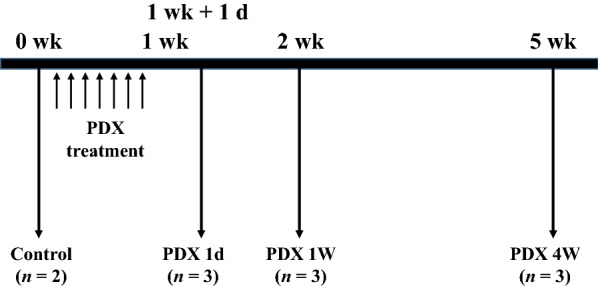
Experimental design

Eleven beagle dogs approximately 2 years of age were used to evaluate PDX-induced neuropathy. The body weight of the dogs ranged from 7 to 11 kg. Two dogs were included in the control group and nine in the experimental group. Three of the dogs in the experimental group were included in the 1-day post-PDX treatment group, three were included in the 1-week group post-PDX treatment group, and three were in the 4 weeks post-PDX treatment group (Fig. 6).

All dogs (purchased from Daeil nongwon, Daeil, KyeongGi, Korea) were clinically judged to be in good health and neurologically normal. All experimental dogs had their own admission number (SNU-091009-1) from the Institute of Laboratory Animal Resources, Seoul National University (Korea). During the experiment, all dogs were cared for in accordance with the Animal Care and Use Guidelines of the Institute of Laboratory Animal Resources, Seoul National University.

### PDX intoxication

PDX (Sigma, St. Louis, MO, USA) was diluted in a 0.9% sterile aqueous solution of sodium chloride and was administered subcutaneously (SC) once a day during the morning for seven consecutive days. The PDX treatment solution was prepared immediately prior to each injection. Animals in the control group received vehicle (iso-osmotic sterile aqueous solution of sodium chloride), while animals in the experimental group were administered 150 mg/kg of PDX in a volume of 100 mg/mL SC [7].

### Body weight measurements and postural reaction assessments

The body weights of the test dogs were measured every morning during the PDX injection period, as well as at 1 week and 4 weeks after the last PDX treatment.

Postural reaction assessments (Table 1) were conducted on all dogs every morning during the PDX injection period, as well as at 1 week and 4 weeks after the last PDX treatment.

**Table 1:** Postural reaction test

Wheel-barrowing test
Hopping test
Hemi-walking test
proprioceptive positioning test

### Electrophysiological recording

All dogs were pre-anesthetized with atropine (0.1 mg/kg of body weight, IM). Anesthesia was induced with diazepam and maintained with isoflurane (Baxter Healthcare, Deerfield, IL) through a semi-closed system. Subcutaneous temperature was maintained at 37–38 °C. A Neuropack2 system (Nihon Koden, Tokyo, Japan) was used for all recordings. All measurements were conducted in the left hindlimb of each dog.

The DEMP, M waves were recorded for the tibial nerve using a 1 Hz, 0.5 ms, supramaximal stimulus. Stimulating electrodes were positioned in the distal tibial nerve and recording electrodes were positioned in the plantar interosseous muscle, with a ground electrode positioned between the stimulating electrode and recording electrode. The recording electrode was a bipolar needle electrode. The REMP, H-reflexes were recorded using 1 Hz, 0.5 ms, submaximal stimulus. The stimulating electrode was positioned in the tibial nerve adjacent to the hook and a recording electrode and ground electrode were positioned in the same site of the tibial nerve where the M wave was measured. As previously described, at least 8 measurements were obtained [7]. Electrophysiological recordings were conducted before the PDX treatment period and at 1 day, 1 week, and 4 weeks after the last PDX treatment.

### Immunohistochemistry

For euthanasia, cephalic veins of the selected dogs underwent intravenous (IV) catheterization. The dogs were anesthetized with a high dose of propofol (5 mg/kg body weight, IV). and tiletamine/zolazepam (10 mg/kg body weight, IV). After confirmation of deep anesthesia, they were perfused transcardially with 0.1 M phosphate-buffered saline (PBS, pH 7.4) and followed by 4% paraformaldehyde in 0.1 M phosphate buffer (PB, pH 7.4). The DRGs of cervical, thoracic, lumbar spinal cords were removed and post-fixed in the same fixative for 12 h, after which they were dehydrated with graded concentrations of alcohol for embedding in paraffin. Next, the paraffin-embedded tissues were sectioned via microtome (Leica, Wetzlar, Germany) into 3 μm coronal sections and the sections mounted onto silane-coated slides. To elucidate the PDX-induced DRG damage, the samples were deparaffinized in xylene, rehydrated in a descending ethanol series, and stained with HE. In addition, CV staining was conducted as previously described [2]. Briefly, CV acetate (Sigma) was dissolved at 1.0% (w/v) in distilled water, and glacial acetic acid was then added to this solution. Before and after staining for 2 min at room temperature, the sections were washed twice in distilled water, dehydrated with graded concentrations of alcohol at room temperature, and finally mounted with Canada balsam (Kanto, Tokyo, Japan). Images of all CV-stained neurons from the DRG were obtained by using a BX51 light microscope (Olympus, Tokyo, Japan) equipped with a digital camera (DP71, Olympus) connected to a personal computer monitor. DRG neurons were separated into three categories according to their sizes: small- (area 1000 μm^2^), medium- (area 1000–2000 μm^2^), and large-sized (> 2000 μm^2^). The number of neurons in the DRG in each group was counted using an image analyzing system equipped with a computer-based CCD camera (software: Optimas 6.5, CyberMetrics, Scottsdale, AZ, USA). Mean cell counts were obtained by averaging the counts from sections taken from each animal. Immunohistochemistry for GFAP and Iba-1 was conducted to elucidate temporal changes in reactive gliosis after PDX treatment. The sections were sequentially treated with 0.3% hydrogen peroxide in PBS for 30 min and 10% normal goat serum in 0.05 M PBS for 30 min, after which they were incubated with diluted rabbit anti-GFAP (1:1000, Chemicon, Temecula, CA, USA) or rabbit anti-Iba-1 antibody (1:500, Wako, Osaka, Japan) overnight at room temperature and subsequently exposed to FITC-conjugated anti-rabbit IgG (1:200; Jackson ImmunoResearch, West Grove, PA, USA) and FITC-conjugated anti-rat IgG (1:600; Jackson ImmunoResearch). The immunoreactions were observed under a BX51 microscope attached to a fluorescent lamp. Analysis of regions of interest in each of the three DRG regions was performed using an image analysis system. Images were converted into an array of 512 × 512 pixels corresponding to a tissue area of 140 × 140 μm (40× primary magnification). Individual pixel resolution included 256 gray levels. The intensity of GFAP and Iba-1 immunoreactivities was evaluated by determining the relative optical density (ROD), which was obtained after the transformation of the mean gray level using the formula: ROD = log (256/mean gray level). The ROD of the image background was determined using NIH Image 1.59 software in unlabeled portions and that value subtracted for correction, thereby yielding high ROD values in areas of preserved structures and low ROD values in areas of structural loss. The ROD ratio is presented as a percentage.

### Statistical analysis

All data are expressed as mean ± SE or mean ± SEM values. Differences in the data were evaluated by applying Student’s *t*-test. Statistical significance was considered present at *P* < 0.05. A paired *t*-test was conducted for analysis of differences in body weights and M wave and H-reflex amplitudes before and after the pharmacologic treatment. The level of significance was set at *P *< 0.05.

## Supplementary information

**Additional file 1: Figure S1.** Hematoxylin and eosin (HE) staining of the dorsal root ganglia (DRG) in the control (A, E, and I) and pyridoxine-treated (B-D, F-H, and J-L) groups. In the control group, neurons in all DRG regions are well-stained by HE. Note that some hematoxylin-stained nuclei have aggregated at one day after the last pyridoxine treatment; thereafter, the level of aggregation decreases with time following the last pyridoxine treatment. Scale bar = 100 μm.

## Data Availability

The datasets used and/or analyzed during the current study including the tissue samples of dorsal root ganglion generated during and/or analyzed during the current study are available from the corresponding author on reasonable request.

## Abbreviations

CV: Cresyl violet
DEMP: Direct-evoked muscle potentials
DRG: Dorsal root ganglia
GFAP: Glial fibrillary acidic protein
HE: Hematoxylin and eosin
Iba-1: Ionized calcium-binding adapter molecule 1
PDX: Pyridoxine
REMP: Reflex-evoked muscle potentials
ROD: Relative optical density
ROI: Region of interest
SGC: Satellite glial cells
