# Supplementary figures and images for: Neuropathological changes in dorsal root ganglia induced by pyridoxine in dogs
Source: BMC Neurosci. 2020 Mar 24;21:11. doi: 10.1186/s12868-020-00559-3 (PMC7092458; doi:10.1186/s12868-020-00559-3)

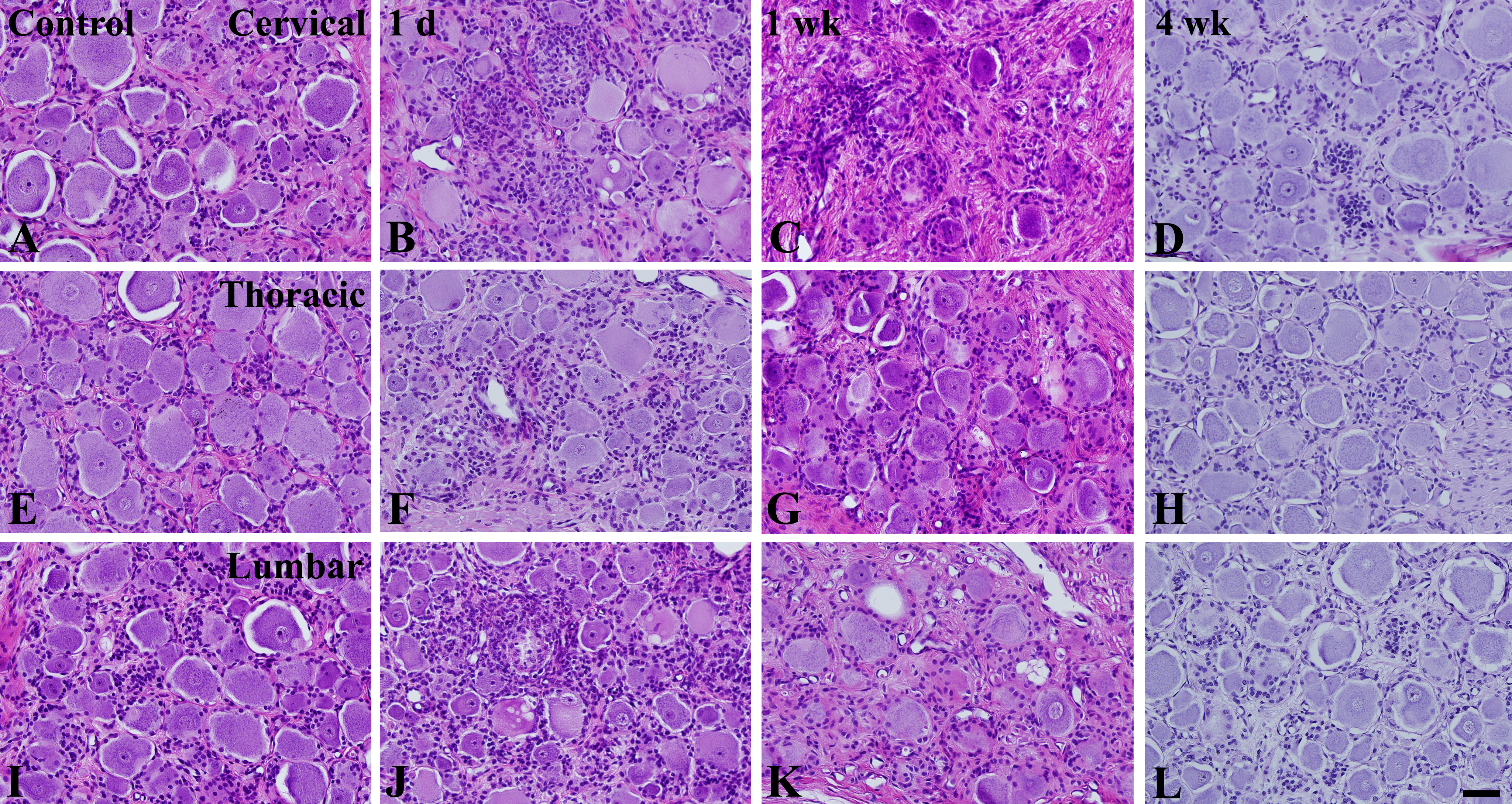

Supplement: Supplementary file 1 — Additional file 1: Figure S1. Hematoxylin and eosin (HE) staining of the dorsal root ganglia (DRG) in the control (A, E, and I) and pyridoxine-treated (B-D, F-H, and J-L) groups. In the control group, neurons in all DRG regions are well-stained by HE. Note that some hematoxylin-stained nuclei have aggregated at one day after the last pyridoxine treatment; thereafter, the level of aggregation decreases with time following the last pyridoxine treatment. Scale bar = 100 μm. [file 12868_2020_559_MOESM1_ESM.tif]
